# Association of WeChat-based transitional care with quality of life and recurrence in patients with acute pancreatitis: A retrospective matched cohort study

**DOI:** 10.12669/pjms.42.6.15536

**Published:** 2026-06

**Authors:** Huaigu Xia, Yanfei Zhang, Dan Lang, Xiaofei Wang

**Affiliations:** 1 Huaigu Xia, Department of Gastroenterology, The First People’s Hospital of Linping District, Hangzhou City, Zhejiang Province 311100, P.R. China; 2 Yanfei Zhang, Department of Gastroenterology, The First People’s Hospital of Linping District, Hangzhou City, Zhejiang Province 311100, P.R. China; 3 Dan Lang, Department of Gastroenterology, The First People’s Hospital of Linping District, Hangzhou City, Zhejiang Province 311100, P.R. China; 4 Xiaofei Wang, Department of Gastroenterology, The First People’s Hospital of Linping District, Hangzhou City, Zhejiang Province 311100, P.R. China

**Keywords:** Acute pancreatitis, Recurrence, Transitional care, Quality of life, WeChat-based

## Abstract

**Objective:**

To evaluate the association of WeChat-based transitional care with quality of life and recurrence in patients with acute pancreatitis (AP).

**Methodology:**

This retrospective study included clinical data from 164 AP patients treated at The First People’s Hospital of Linping District, Hangzhou from July 2023 to June 2024. Of them, 82 patients received WeChat-based transitional care after discharge (observation group). In addition, a control group of 82 patients with AP who received conventional standard care from June 2022 to June 2023 was selected and matched with the observation group at a 1:1 ratio according to sex and age. The Chinese version of the Short Form 36 Health Survey (SF-36) was used to assess patients’ quality of life and the 1-year recurrence rate.

**Results:**

At six months after discharge, the scores of all dimensions of the SF-36 in both groups were significantly higher than those before intervention, and the scores of all dimensions in the observation group were significantly higher than those in the control group (P < 0.05). After one year of follow-up, the observation group had a lower recurrence rate than the control group [11.0% (9/82) vs. 24.4% (20/82), P < 0.05]. The Kaplan-Meier analysis showed that the recurrence-free survival time in the observation group was longer than that in the control group (*χ^2^* = 4.829, P = 0.028).

**Conclusions:**

WeChat-based transitional care was associated with better quality-of-life outcomes and a lower recurrence rate in patients with AP. Given the retrospective matched design, these findings should be interpreted as preliminary associations and require confirmation in future prospective studies.

## INTRODUCTION

Gallstones and alcohol consumption remain the primary etiological factors of acute pancreatitis (AP), a major cause of hospitalization for gastrointestinal disorders.^1^ Reducing recurrence and improving long-term outcomes through systematic management after discharge has become an important extension of AP management. Recurrent AP (RAP) increases the risk of readmission and associated medical costs, is often associated with persistent symptoms, local complications, or disease recurrence, and may promote progression to chronic pancreatitis.^2^–^4^ Therefore, individualized discharge plans during the transition period from hospital discharge to home care are critical and may directly impact recurrence and readmission.

In addition to event-based outcomes, quality of life (QoL) is also a core indicator for the long-term management of AP. The condition is often characterized by persistent pain, and impacts general health perception, vitality, and mental health of patients.^5^ Psychometric studies have also emphasized the substantial subjective burden of pancreatic diseases. The pancreatic disease-specific QoL tool developed and validated by Wassef et al.^6^ suggests that physiological indicators alone cannot fully reflect patients’ experiences. Therefore, incorporating QoL into the assessment of post-discharge management helps to more comprehensively measure the benefits of interventions.

While continuous care often relies on information handover, health education, and self-management support, there are still considerable communication gaps between inpatient care and primary care/family settings. Kripalani et al.^7^ noted that insufficient information transfer between in-hospital physicians and primary care providers may endanger patient safety and undermine continuous care. A systematic review and meta-analysis by Becker et al.^8^ also suggested that interventions targeting discharge communication are closely associated with patient outcomes such as readmission. WeChat, widely used in China, is a fast, lightweight, and extensible text-based chat client. It offers a broad population reach, real-time interactive communication, and support for multimedia-based health education. Multimodal WeChat-based nursing programs have been shown to improve health-related QoL, confirming their feasibility and effectiveness.^9^ However, evidence regarding WeChat-based transitional care for post-discharge AP patients with recurrence-QoL as the core outcomes remains limited.

This matched controlled retrospective cohort study evaluated the association of WeChat-based transitional care combined with follow-up management with post-discharge QoL, measured using the Chinese version of the SF-36 Health Survey, and 1-year recurrence risk in patients with AP. The study aimed to provide preliminary evidence for the potential role of digital transitional nursing in post-discharge AP management and to explore whether this model was associated with better long-term health status and a lower recurrence risk.

## METHODOLOGY

This retrospective matched comparative study included patients with acute pancreatitis (AP) treated at The First People’s Hospital of Linping District, Hangzhou. Patients who received WeChat-based transitional care after discharge from July 2023 to June 2024 were assigned to the observation group, whereas those who received conventional standard care from June 2022 to June 2023 were selected as historical controls. A 1:1 individual matching approach based on sex and age was used to improve baseline comparability. For each patient in the observation group, one patient from the historical control period was selected according to the same sex and similar age. This was not a propensity score matching procedure; therefore, propensity scores were not calculated and no caliper was applied. Post-matching comparability was assessed descriptively by comparing baseline characteristics between the two groups, as shown in Table-I. During the study period, the core inpatient treatment protocol for AP, routine nursing team structure, basic discharge education pathway, and electronic medical record workflow remained generally consistent in our department. No parallel departmental quality-improvement program specifically targeting AP recurrence or post-discharge quality of life was implemented during these periods. The main planned difference between the two periods was the addition of WeChat-based transitional care and follow-up management in the observation group.

**Table-I:** Basic characteristics of patients.

Characteristics	Observation group (n=82)	Control group (n=82)	χ^2^/t	P
Male n(%)	46 (56.1)	51 (62.2)	0.631	0.427
Age (year)	55.6±9.3	53.9±10.7	1.139	0.256
Marital status, n(%)			0.425	0.515
Married	71 (86.6)	68 (82.9)		
Unmarried or widowed	11 (13.4)	14 (17.1)		
Degree of education, n(%)			3.543	0.17
Junior high school and below	31 (37.8)	39 (47.6)		
High school	31 (37.8)	32 (39.0)		
College degree or above	20 (24.4)	11 (13.4)		
Per capita household income (yuan/month), n(%)			0.681	0.711
<4000	32 (39.0)	27 (32.9)		
4000-6000	33 (40.3)	37 (45.1)		
>6000	17 (20.7)	18 (22.0)		
Etiology, n(%)			2.876	0.411
Biliary origin	47 (57.3)	40 (48.8)		
Alcoholic	13 (15.9)	21 (25.6)		
Hyperlipidemic type	16 (19.5)	17 (20.7)		
Others	6 (7.3)	4 (4.9)		
Diabetes, n(%)	10 (12.2)	16 (19.5)	1.645	0.2
Hypertension, n(%)	19 (23.2)	21 (25.6)	0.132	0.716
Smoking, n(%)	37 (45.1)	33 (40.2)	0.399	0.528

### Ethical Approval:

This study was approved by the Ethics Committee of The First People’s Hospital of Linping District before the start of data analysis (No. 2023-068; June 20, 2023). Because this was a retrospective study based on existing clinical and follow-up data and did not involve additional procedures beyond routine clinical management, the requirement for written informed consent was waived by the ethics committee. All patient information was de-identified before analysis.

The diagnosis of AP was based on the commonly used clinical criteria, requiring the presence of at least two out of the following three items:

- Typical epigastric pain.
- Serum amylase and/or lipase levels elevated to more than three times the upper limit of normal.
- Imaging findings suggestive of pancreatitis changes.

All study participants met the discharge criteria after inpatient treatment and completed post-discharge follow-up management.

### Inclusion criteria:

- Discharge after meeting the diagnostic criteria for AP and completing hospitalization treatment.
- Age ≥ 18 years old.
- Having stable follow-up conditions (follow-up can be completed through WeChat/phone).
- The clinical data are complete, including those who completed the intervention.

### Exclusion criteria:

- Patients with concurrent malignant tumors or significantly limited expected survival.
- Patients with a history of chronic pancreatitis or pancreatic resection that may significantly affect their QoL assessment.
- Patients with severe mental/cognitive impairments or inability to cooperate with follow-up assessments.
- Patients with missing or lost follow-up data after discharge.

### Intervention Measures:

All members of the follow-up management team underwent standardized training, and follow-up manuals were used. Regular assessments of patients’ medical conditions and lifestyle changes and their adherence to follow-up protocol were conducted via WeChat questionnaires or online communication. Identifiers were removed for patients throughout the entire intervention process.

### Conventional Standard Care/Routine Follow-up:

Patients in the control group received routine treatment and nursing care during hospitalization. Routine discharge guidance was provided upon discharge, including medication instructions, dietary recommendations, follow-up appointment reminders, and notification of warning signs. After discharge, patients attended routine outpatient follow-ups or received telephone follow-ups based on standard clinical practice, without a fixed online management protocol.

### WeChat-based Transitional Care and Follow-up Management:

In addition to the routine discharge guidance, transitional care via the WeChat platform was implemented, primarily delivered by a follow-up management team led by specialist nurses and collaborating with attending physicians. The core components of the intervention were as follows:

### File Establishment and Group Enrollment:

Prior to discharge, a WeChat-based follow-up file was created for each patient, containing basic information, etiology type, key laboratory/imaging results, post-discharge medication plans, and dietary recommendations. Patients were instructed by nurses to join the designated WeChat follow-up channel, which could be either one-on-one communication or a unified follow-up group, depending on practical implementation. The contact frequency was basically fixed according to the follow-up protocol. For privacy and data-security protection, WeChat-based communication was managed only by authorized members of the follow-up team. Patient follow-up files were maintained using study codes or follow-up identifiers whenever possible. Sensitive medical information was not disclosed publicly in group communication, and individualized medical issues were addressed through one-to-one communication when necessary. The research dataset was stored in a de-identified form, and access was restricted to the study team.

### Health Education and Behavioral Guidance:

Structured education with standardized content was provided focusing on key post-discharge management priorities for AP, including the principles of low-fat diet and meal fractionation, alcohol abstinence and high-risk behavior control, blood lipid/glucose management, regular follow-up and examinations, as well as identification of warning symptoms (such as recurrent abdominal pain, fever, vomiting) and corresponding medical consultation pathways. Educational materials were delivered in multiple formats, such as graphic push notifications, short videos/voice prompts, and key-point checklists.

### Symptom Monitoring and Q&A Support:

Patients’ symptoms and adherence to lifestyle recommendations (such as abdominal pain, nausea and vomiting, abdominal distension, bowel movements, dietary tolerance) were collected regularly via WeChat by nurses, and risk-stratified management was used for symptoms. Immediate guidance was provided by physicians for abnormal patient-reported symptoms, and outpatient follow-up or emergency evaluation was recommended when necessary.

### Follow-up Reminders and Medication Management:

Reminders for follow-up appointments, prompts for medication adherence, and education on adverse drug reactions were delivered in accordance with post-discharge medical orders. Individualized management priorities were reinforced for patients with high-risk factors (such as hypertriglyceridemia, history of alcohol consumption).

### Psychological Support and Rehabilitation Promotion:

Attention was paid to patients’ anxiety levels, sleep quality, and recovery of social function. Psychological support and lifestyle adjustment recommendations were provided to facilitate patients’ return to daily life and work.

The intervention lasted for at least six months, and follow-up was continued for one year after discharge to assess recurrence outcomes.

### Observation indicators and evaluation methods:

### QoL assessment:

The Chinese version of the short-form Health Survey (SF-36) is a validated tool for measuring health-related QoL in Chinese populations. In the present study, it was used to evaluate the QoL. SF-36 consists of eight dimensions: Physical Function (PF), Physical Role (RP), Physical Pain (BP), Overall Health (GH), Vitality (VT), Social Function (SF), Emotional Role (RE), and Mental Health (MH). Scores for each dimension range from 0 to 100, with higher scores indicating better QoL. The evaluation time points are one day before discharge (baseline) and six months after discharge (follow-up).

### Recurrence outcome:

Recurrence was defined as the recurrence of AP attacks that meet the diagnostic criteria for AP during the follow-up period after discharge (based on medical records of re-visit/readmission, laboratory tests, and imaging data). The recurrence rate was calculated within one year after discharge, and the time of first recurrence was recorded for recurrence-free survival analysis (Kaplan-Meier).

### Baseline data collection:

General patient information and clinical features were extracted from the electronic medical record system, and included gender, age, etiology type, severity assessment information, major comorbidities, hospitalization treatment, and complication records.

### Follow-up method:

The observation group mainly completed follow-up and data collection via WeChat, with telephone follow-up as needed; the control group reported outcome information through routine outpatient follow-up or telephone follow-up. SF-36 was completed under medical staff guidance one day before discharge. Six months after discharge, patients were guided via WeChat or phone to complete the scale assessment. The recurrence event was verified through follow-up records and in-hospital visits/readmission medical records, with follow-up up to one year after discharge.

### Statistical methods:

Statistical software SPSS version 26.0 (IBM Corp, Armonk, N.Y., USA) was used for data analysis. Measurement data were expressed as mean ± standard deviation or median (interquartile range, M [IQR]) based on distribution. Independent sample *t*-test or Mann-Whitney *U* test were used for intergroup comparison, while paired t-test or Wilcoxon signed rank test was used for intragroup comparison before and after. Count data were presented in terms of examples (percentage), and comparison between groups is performed using the chi-square test or Fisher’s exact test. Event-free survival time to recurrence was estimated using the Kaplan-Meier method, and the difference between the two groups was assessed using the Log-rank test. Bilateral test, P<0.05 indicates a statistically significant difference.

## RESULTS

This retrospective analysis included clinical data of 164 AP patients (97 males and 67 females) with ages ranging between 26 and 75 years (an average age of 54.7 ± 10.0 years). A total of 82 patients who received WeChat-based transitional care were matched at a 1:1 ratio with 82 patients who received conventional standard care. There was no statistically significant difference in the basic characteristics between the two groups (P>0.05) (Table-I).

On the first day prior to discharge, there were no significant differences in the scores of all SF-36 dimensions between the two groups (P>0.05). At six months post-discharge, the scores of all SF-36 dimensions in both groups significantly increased, and the scores of all dimensions in the observation group were considerably higher than in the control group (P<0.05) (Table-II).

**Table-II:** Comparison of SF-36 scores in various dimensions between two groups of patients.

Variables	Observation group M (IQR) (n=82)	Control group M (IQR) (n=82)	P
***One Day Before Discharge***			
Physical Functioning	81 (74-85)	84 (78-87)	0.068
Role-Physical	62 (56-68)	64 (57-68)	0.288
Bodily Pain	52 (48-58)	52.5 (47-59)	0.579
General Health	63.5 (58-69)	62.5 (56-66)	0.149
Vitality	63 (58-68)	62 (56-67)	0.155
Social Functioning	62.5 (57-70)	60.5 (56-64)	0.267
Role-Emotional	56.5 (52-62)	54 (52-58)	0.131
Mental Health	64 (58-72)	63 (54-69)	0.167
***Six Months Post-Discharge***			
Physical Functioning	92 (87-96)	89 (84-93)	0.007
Role-Physical	79 (73-86)	75 (70-82)	0.002
Bodily Pain	84 (75-86)	78 (69-83)	<0.001
General Health	86 (79-91)	76.5 (74-82)	<0.001
Vitality	82.5 (75-85)	78 (72-84)	0.012
Social Functioning	85 (79-89)	78 (69-84)	<0.001
Role-Emotional	75 (68-83)	70 (65-78)	0.001
Mental Health	86 (77-89)	77 (71-84)	<0.001

**Fig.1:**
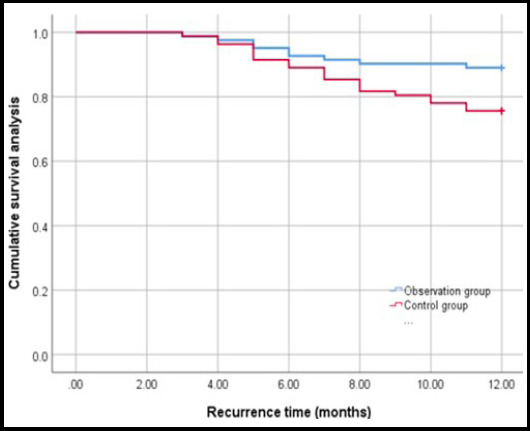
Recurrence-Free Survival Curve.

During the one-year follow-up period, the recurrence rate in the observation group was 11.0% (9/82), significantly lower than that of the control group [24.4% (20/82)] (*χ^2^*=5.069, P<0.05). Kaplan-Meier analysis showed that the recurrence-free survival time in the observation group was longer than that in the control group (*χ^2^*=4.829, P=0.028) (Fig.1).

## DISCUSSION

The results of this matched-control, retrospective study demonstrated that, compared with routine follow-up, WeChat-based transitional care further improved scores on all SF-36 dimensions in patients with AP at six months after discharge. During the one-year follow-up, the WeChat group had a lower recurrence rate and longer recurrence-free survival. These findings suggest that WeChat-based transitional care bridging in-hospital treatment and post-discharge management is associated with better patients’ subjective health status and a lower disease recurrence risk by enabling early risk identification and enhancing behavioral management.

The observed improvement in QoL associated with WeChat-based care is consistent with previous evidence. A systematic review and meta-analysis focusing on QoL after AP indicated that, following resolution of the acute phase, patients may still experience persistent impairments in physical function and mental health, as well as higher pain levels, and that QoL recovery does not rely entirely on spontaneous healing.^10^ In this study, the improvement in SF-36 scores observed in both groups aligns with the typical rehabilitation trajectory, while the greater magnitude of improvement in the observation group suggests that WeChat-based transitional care provides additional benefits in symptom management, psychological support, and lifestyle recovery. Psychometric studies also support the importance of prioritizing patient-reported outcomes (PRO). A study by Wassef et al.^6^ showed that subjective burdens associated with pancreatic diseases, such as pain, functional limitations, and emotional distress, are key outcome dimensions. Wehler et al.^11^ emphasized that QoL is influenced by multiple factors and has a measurable, stable structure. Therefore, the use of the SF-36 in this study is both comparable and practical, and suggests that future studies could combine it with more pancreas-specific PRO tools to capture more subtle changes.^6^,^11^ Previous surveys of discharged patients with severe AP have indicated that pain control, nutritional status, complication burden, and family/social support are closely correlated with long-term QoL.^12^ WeChat-based transitional care can transform discharge education into continuous, traceable support. It strengthens self-management through regular information push, reminders, and online Q&A, and delivers timely interventions for issues such as anxiety, sleep disturbances, or poor dietary adherence. This may explain the observed positive impact of the WeChat-based intervention on the long-term QoL, and the additional improvements observed in the psychological and social function dimensions in this study.^12^ Improvements in psychological and social function may suggest that WeChat-based communication alleviated patients’ anxiety regarding recurrence and strengthened their confidence during recovery. Furthermore, as some AP patients may develop exocrine pancreatic insufficiency (EPI), which can lead to malnutrition, weight loss, and other complications that affect recovery quality, clinical updates from the American Gastroenterological Association (AGA) emphasize key long-term management points, including EPI symptom recognition, nutritional assessment, and pancreatic enzyme replacement therapy.^13^ Thus, integrating digestive symptom monitoring and nutritional risk screening into WeChat follow-up interventions may represent an important pathway to improve physical health and vitality.^13^ Improvements in physical health may indicate the recovery of the ability to perform daily activities.

This study also showed that the WeChat-based intervention was associated with a lower one-year recurrence risk, consistent with the existing evidence that behavioral factors are modifiable and that etiological management requires continuity. For instance, for alcohol-related AP, intensive education during hospitalization can significantly reduce post-discharge drinking behavior.^14^ Randomized controlled trials have also indicated that systematic interventions can decrease recurrence of alcohol-related AP.^15^ It is plausible that WeChat-mediated follow-up extends the management of alcohol abstinence, lipid control, and dietary structure optimization into the post-discharge period and improves adherence through continuous reminders and interactive feedback, consistent with previous studies.^14^,^15^ In hypertriglyceridemic AP, readmission risk is closely associated with metabolic control,^16^ suggesting that continuous supervision of diet, body weight, and lipid management during the post-discharge phase may be particularly critical. At the same time, some structural/pancreatobiliary-related etiologies of recurrent pancreatitis require endoscopic evaluation and treatment.^17^ WeChat-based management does not replace etiological treatment; instead, it may facilitate earlier identification of recurrence patterns and promote timely referral, enabling patients to access standardized assessment and treatment pathways more quickly, thus complementing specialist interventions.^17^

From the perspective of post-discharge to home transitional care management, the findings of this study are consistent with readmission prevention and control strategies. Reviews focusing on reducing hospital readmissions demonstrated the importance of integrated strategies, including discharge planning, early follow-up, medication/symptom education, and multidisciplinary collaboration.^18^ Similarly, a study by Metra et al.^19^ highlighted the core roles of pre-discharge assessment, early post-discharge follow-up, and symptom monitoring in patients with heart failure. A randomized controlled trial by Wang et al.^20^ demonstrated that multimodal digital management via WeChat could improve management outcomes in patients with newly diagnosed mild-to-moderate hypertension. In pancreatic-related diseases, real-time doctor-patient communication via WeChat has also been reported to correlate with clinical outcomes, suggesting its potential to enhance follow-up efficiency and communication quality.^21^ Since the concept of continuous transitional care is generally transferable across disease entities, these results collectively further demonstrate that WeChat is an efficient technical platform for high-frequency patient engagement and low-cost follow-up, providing external validation of the reduced recurrence rate observed in the present study.

In addition, studies on telerehabilitation and nutritional management in other disease areas have indirectly verified the value of sustained intervention during the home care phase. A systematic review of home-based telerehabilitation for swallowing disorders in head and neck cancer patients concluded that this model is feasible and may improve functional outcomes.^22^ Perioperative exercise programs have been shown to enhance physical fitness in patients with esophageal/gastric cancer.^23^ Nurse-led individualized mobile health (mHealth) nutritional interventions can improve nutrition-related outcomes and promote recovery in post-discharge patients after gastric cancer surgery.^24^ These studies suggest the effectiveness of multi-module, feedback-enabled home-based interventions for improving long-term outcomes, and provide indirect evidence for the integration of exercise, nutrition, and psychological support modules into the care of AP patients.^22^–^24^

### Strengths of the study:

The SF-36 has been reported to be useful in general populations in Hangzhou, China.^25^ The present study simultaneously incorporated the SF-36 scale and recurrence/recurrence-free survival time as outcomes, addressing both patient-reported experiences and clinical events. The intervention was delivered via the WeChat platform, which offers low cost, high accessibility, ease of standardized information delivery, and traceable management, thus providing preliminary evidence supporting a digital transitional care model in AP. Finally, it integrated QoL improvement and recurrence prevention into a single continuous care framework, thereby strengthening the operability of post-discharge management strategies.^18^–^20^

### Limitations:

First, it was a single-center retrospective study using sequential historical controls, which may introduce selection bias and temporal bias. Although no major documented changes were identified in the core inpatient treatment protocol, routine nursing team structure, discharge education pathway, or electronic medical record workflow during the study period, gradual improvements in staff experience, patient education quality, early recurrence recognition, or other unmeasured workflow-related factors may still have influenced the results. Therefore, the findings should be interpreted as associations rather than definitive causal effects, and their generalizability remains limited. Second, although 1:1 individual matching by sex and age was used, this was not a propensity score matching procedure. Propensity scores were not calculated, no caliper was applied, and formal balance assessment using standardized mean differences or a Love plot was not performed. Residual imbalance in measured or unmeasured confounders, including disease severity, etiological treatment completion, metabolic control, comorbidity burden, and post-discharge adherence, cannot be fully excluded. Third, multivariable Cox regression was not performed because only 29 recurrence events occurred during the one year follow up period, which limited the reliability of a multivariable model and increased the risk of overfitting. In addition, detailed information on etiology-specific treatment was incomplete because some key treatments, such as cholecystectomy or ERCP, alcohol abstinence, and long-term lipid or metabolic control, may have occurred after discharge or at other hospitals and were not uniformly recorded. Therefore, residual confounding related to etiological management remains possible. Finally, subgroup analyses by etiological subtype or structural causes were insufficient, and the SF-36, as a generic quality-of-life instrument, may not fully capture AP-specific recovery issues such as dietary tolerance, digestive discomfort, exocrine pancreatic insufficiency, weight change, or recurrence-related anxiety.

## CONCLUSION

In this retrospective matched cohort study, WeChat-based transitional care was associated with better SF-36 scores, a lower recurrence rate, and longer recurrence-free survival during the 1-year follow-up period among patients with AP after discharge. These findings provide preliminary evidence supporting a digital transitional care model in AP; however, prospective multicenter randomized studies are needed to confirm its effectiveness and clarify the contribution of individual intervention components.

### Recommendations:

Future large-scale, multicenter, prospective randomized studies should standardize the collection of etiological treatment, metabolic control, adherence, recurrence, readmission, cost-effectiveness, and disease-specific patient-reported outcome data to confirm these findings and optimize the WeChat-based transitional care model.

### Author’s contributions:

**HX:** Literature search, study design and manuscript writing.

**YZ, DL and XW:** Data collection, data analysis and interpretation. Critical Review.

**HX:** Manuscript revision and validation and is responsible for the integrity of the study.

All authors have read and approved the final manuscript.
